# Atrial electromechanical delay and p wave dispersion associated with severity of chronic obstructive pulmonary disease

**DOI:** 10.4314/ahs.v21i1.19

**Published:** 2021-03

**Authors:** Yunus Celik, Nesligül Yıldırım, Vahit Demir, Cağlar Alp, Omer Sahin, Mehmet Tolga Doğru

**Affiliations:** 1 Department of Cardiology, Kırıkkale Yuksek Ihtisas Hospital, Kırıkkale, Turkey; 2 Department of Cardiology, Medical Faculty of Kırıkkale University, Kırıkkale, Turkey; 3 Department of Cardiology, Medical Faculty of Bozok University, Yozgat, Turkey; 4 Department of Cardiology, Medicana Sivas Hospital, Sivas, Turkey

**Keywords:** Atrial eletromechanical delay, chronic obstructive pulmonary disease, P wave dispersion

## Abstract

**Background:**

The aim of this study was to evaluate atrial electromechanical delay (AEMD) with both electrocardiography (ECG) and echocardiography in patients with Chronic Obstructive Pulmonary Disease (COPD).

**Methods:**

Total of 110 patients were included in this cross-sectional case-control study. P-wave dispersion (PWD) was measured on a 12-lead ECG. Atrial electromechanical intervals (PA) were measured as the time interval between the onset of the P wave on the ECG and the beginning of the late diastolic A wave.

**Results:**

PWD was found to be 40.9±9.2 ms in the healthy control group, 45.6±8.2 ms in the mild COPD and 44.8±8.7 ms in the severe COPD group (p<0.05). Intra-right atrial EMD was found to be 10.7±5.8 ms in mild COPD, 11.0±7 ms in severe COPD, and it was 16.4±7.3 ms in healthy control group (p<0.001). Interatrial EMD was detected to be 29.5±9.1 ms in the control group, 24.1±9 ms in mild COPD group, and 23.9±11.1 ms in the severe COPD group (p<0.001).

**Conclusion:**

Both mild and severe COPD groups decreased PWD, increased tricuspid PA and significantly decreased interand right intra-AEMD times in comparison to the control group.

## Introduction

Prolonged atrial conduction time increases the risk of various atrial tachyarrhythmia, especially atrial fibrillation (AF). The main reason is that intra and interatrial conduction disorders lead to extended atrial conduction times and cause re-entry circuits^1^,^2^. The mechanisms that initiate AF and cause it to be permanent are not yet known^3^. The studies have shown that atrial conduction disorder may have a role in the initiation and persistence of AF^4^, ^5^. The patients with deteriorated atrial conduction times are determined to have frequent AF attacks^6^. Various methods are being used to assess atrial conduction disorders. The most common methods used for this purpose are electrocardiography (ECG) and transthoracic echocardiography.

Determination of electromechanical incidents via transthoracic echocardiography may be helpful for the calculation of interatrial conduction time. Atrial electromechanical delay (AEMD) can be calculated as the time between the beginning of the P wave in single derivation surface ECG and the atrial contraction determined via tissue Doppler echocardiography^8^–^11^. The measurement of atrial conduction delays using the tissue Doppler method can be used as a non-invasive atrial substrate assessment method. This may be an appropriate method to estimate the risk of developing AF.

Chronic Obstructive Pulmonary Disease (COPD) is characterized by chronic and progressive airflow restriction due to chronic bronchitis and emphysema^12^. Due to the close anatomical and functional relationship between the heart and lungs, it is very natural to have some consequences in case of cardiac or pulmonary dysfunction. Concomitant cardiac diseases increase mortality and morbidity rates in COPD^13^. Therefore, determining the risk of developing atrial rhythm disorders such as AF in the monitoring of COPD will play an important role in the prognosis of the disease.

The purpose of our study is to assess P wave dispersion (PWD) and atrial conduction times in mild and severe stage COPD.

## Material and methods

### Study population

In the cross-sectional study; 200 patients who applied to the Cardiology Department of Kırıkkale University Faculty of Medicine between February 2012 and December 2012 with COPD and related symptoms were examined. A total of 110 patients (20 females, 90 males) between the ages of 42–72, consisting of 35 mild-stage COPD and 35 severe-stage COPD staged according to the 2011 criteria (Global Initiative for Global Obstructive Pulmonary Disease)^14^ and 40 healthy controls were included in the study. The control group was randomly selected from the pool of healthy subjects who applied to the cardiology outpatient clinic who were similar to the patient group in terms of age and gender, and had no COPD diagnosis and received normal test results on transthoracic echocardiography. Patients with COPD attacks in the past month, with uncontrolled hypertension, with left ventricular ejection fraction (EF) <50%, patients with diabetes mellitus, significant valve abnormalities, history of coronary artery disease, drug history that may affect heart rate and hyperthyroidism were excluded from the study. Other exclusion criteria were presence of left bundle branch block, permanent pacemaker implantation, structural heart disease, and active inflammation. Demographic data of all patients were collected and pulmonary function tests (FEV1: exhaled volume in the first second of mandatory expiration, FVC: Forced expiration capacity) and transthoracic echocardiography and ECG measurements were made after a 15-minute rest. The approval of the ethics committee of Kırıkkale University was obtained for this study (Approval no: 12.01.2012-12/06). All subject groups understood the purpose and process of the study and gave written informed consent before participating in the study.

### Twelve-lead resting electrocardiography

The 12-lead surface ECG of all included patients was measured in a supine position. ECG was recorded with recording rate of 25 mm/sec and 20 mm/mV amplitude standards. Measurement results were calculated using the average of three waves examined with each lead. The beginning of the P wave was accepted as the point where the first deflection of the P wave had left the isoelectric line, and the end of the P wave was accepted as the point where the P wave had re-intersected with the isoelectric line. Pmax was measured as the time of the longest P wave within 12 leads, and Pmin was measured as the time of the shortest P wave within 12 leads. The difference between Pmax and Pmin in 12-lead was calculated as P-wave dispersion (PWD) (PWD=Pmax-Pmin). Measurements were conducted manually using a magnifying glass^11^.

### Transthoracic echocardiography

Standard two-dimensional and M mode echocardiography and tissue Doppler echocardiography was performed for all patients. All echocardiography assessments and measurements were conducted as the patient was lying in a left lateral decubitus position and by using Vivid Pro7 echocardiography device (General Electric, Vingmed, Horten, Norway). The calculation of systolic pulmonary artery pressure (SPAP) was performed by considering the continuous wave (CV) Doppler parallel to tricuspid valve insufficiency flow^15^. Left atrium (LA), right atrium (RA), left ventricular posterior wall (LVPW) thickness, left ventricle interventricular septum (LVIVS) thickness, left ventricle end-diastolic diameter (LVEDD) and left ventricle end-systolic diameter (LVEDS) was calculated by using the M-mode method. The Modified Simpson method was used to calculate the left venctricle ejection fraction (EF). The tissue Doppler spectral images were obtained by placing the the tissue Doppler pulsed wave to left ventricle lateral mitral anulus, septal mitral anulus and right ventricle tricuspid annulus respectively in apical four-cavity image window. Based on this, the first negative myocardial velocity started during diastole was named as E' wave and the second negative myocardial velocity was named as A' wave. The E' wave formed at the atrioventricular passive filling phase during diastole, whereas, the A' wave formed after diastatis but with the atrial contraction before the ventricular systolic myocardial movement. The first low positive amplitude myocardial velocity observed during systole was an isovolumic contraction velocity (IVC) wave; the second long wave with a positive high amplitude was called myocardial velocity (S') during ventricular ejection time (ET), and the negative low amplitude rate observed before diastole was called isovolumetric relaxation time (IVRT)^16^.

### Measurement of atrial electromechanical times

In an apical 4-chamber view, the pulsed-wave tissue Doppler imaging signal was recorded with the sample volume placed at the lateral mitral annulus, septal mitral annulus, and right ventricular tricuspid annulus. The time between the beginning of the P wave in ECG and the beginning of A' wave in tissue Doppler traces was defined as the electromechanical delay (PA). Atrial electromechanical delays in all three areas were measured with this method. The difference between the PA times measured from the left ventricle lateral mitral anulus and right ventricle tricuspid anulus areas was defined as echocardiographic interatrial conduction delay (Interatrial EMD), the difference between the PA times measured from left ventricle lateral mitral anulus and septal mitral anulus was defined as the echocardiographic intra-left atrial conduction delay (Intra-left atrial EMD), and the difference between the PA times measured from septal mitral anulus and right ventricle tricuspid anulus was defined as echocardiographic intra-right atrial conduction delay (Intra-right atrial EMD)^17^ (Figure 1).

**Figure:**
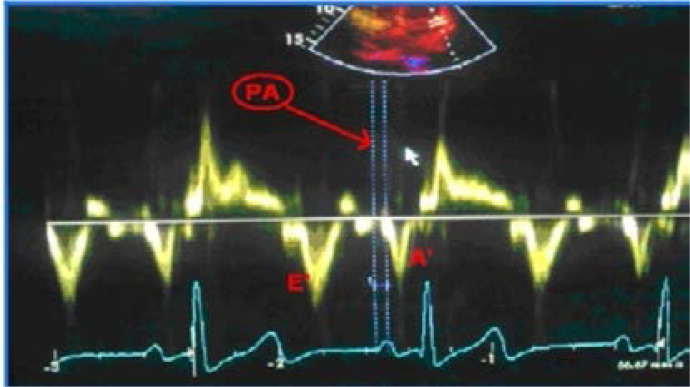
PA: Measurement of time interval from the onset of P-wave on surface electrocardiogram to the beginning of A-wave interval with tissue Doppler imaging.

### Statistical analysis

All statistical analyzes were done using SPSS version 20.0 (SPSS; Chicago, IL, USA). Normally distributed data were expressed as mean ± standard deviation (SD) and non-normally distributed data were expressed as median (25–75%). For continuous data Student t and One-Way ANOVA tests with Bonferroni adjustment were used for comparing normally distributed data. Mann-Whitney U and Kruskal Wallis tests were used for comparing non-normally distributed data. Pearson, Spearman's and Partial correlation analysis tests were utilized for correlation analysis. A p value of <0.05 was accepted as statistically significant.

## Results

Demographic data and pulmonary function test results of patients and control group are summarized in Table 1.

**Table 1:** Demographic characteristics and respiratory function test results of the groups

		Control Group (n:40)	Mild stage COPD (n:35)	Severe stage COPD (n:35)	p*
**Age** (years)		54.95±6.25	55.34±7.20	57.20±5.84	0.28
**Sex**	Female (n)	7	5	8	0.64**
	Male (n)	33	30	27	
**Smoking**	No (n)(%)	22 (%55)	3 (%8.6)	6 (%17)	**<0.001** **
	Yes (n)(%)	18 (%45)	32 (%91)	29 (%82.9)	<0.001**
**BSA** (m^2^)		1.85±0.14	1.88±0.13	1.82±0.19	0.28
**BMI** (kg/m^2^)		28.77±4.55	27.5±3.85	26.40±6.14	0.11
**Systolic BP** (mmHg)		132.45±7.2	134.94±5.5	132.60±9.2	0.29
**Diastolic BP** (mmHg)		80±4.89	80.5±4.7	79.74±7.2	0.82
**FEV1** (%)		93.0±6.03^a^,^b^	64.40±6.6^a^,^c^	39.77±6.7^b^,^c^	**<0.001**
**FVC** (%)		97.7±7.4	89.48±9.74	66±12.9	**<0.001**
**FEV1/FVC** ***		87(80–92)^d^,^e^	64(61–68)^d^,^f^	61(57–63)^e^,^f^	**<0.001**

* One-wayAnova test

** Chi-square test

*** Kruskal Wallis test

Oneway ANOVA test, Mean±SD, p<0.05, Kruskal-Wallis Test, Median (%25–%75), p<0.05

BSA: Body surface area, BMI: Body mass index, BP: Blood pressure, FEV1: The volume exhaled at the first second of forced expiration, FVC: Forced expiration capacity

a Control group-mild stage COPD (p=<0.001)

b Control group–severe stage COPD (p=<0.001)

c Mild stage COPD-Severe stage COPD (p=<0.001)

d Control group-mild stage COPD (p=<0.001)

e Control group-severe stage COPD (p=<0.001)

f Mild stage COPD-severe stage COPD (p=0.001)

There was no significant difference between these two groups in terms of age, body surface area, body mass index, systolic and diastolic blood pressure. Although there was no gender difference, there was male domination in both groups. As expected, there were more smokers in the patient groups than in the control group. There was a significant difference between the groups for FEV1, FVC and FEV1 / FVC parameters (p = <0.001). The heart rates, Pmax and Pmin values of the ECG are shown in Table 2.

**Table 2:** Electrocardiographic parameters

Parameters	Control Group (n:40)	Mild Stage COPD (n:35)	Severe Stage COPD (n:35)	p*
**Pace** (pulse/min)	73.8±9.0	72.9±8.3	77.7±11.4	0.09
**Pmax** (ms)	92.0±16.48	97.60±12.09	98.05±15.22	0.14
**Pmin** (ms)	51.05±12.46	51.94±10.59	53.17±10.38	0.71
**PWD** (ms)	40.9.5±9.2^a^,^b^	45.65±8.20^a^,^c^	44.88±8.73^b^,^c^	**0.04**

* One-wayAnova test

Oneway ANOVA test, Mean±SD, p<0.05, *Kruskal-Wallis Test, Median (%25–%75),p<0.05

Pmax: Longest P wave duration, Pmin: Shortest P wave duration, PWD: P wave dispersion

a Control group-mild stage COPD (p=0.034)

b Control group–severe stage COPD (p=0.042)

c Mild stage COPD-severe stage COPD (p= 0.347)

Also a significant difference was found between the control group and both mild-stage COPD and severe COPD groups in terms of PWD (p = 0.034, p = 0.042, respectively). The conventional echocardiographic measurements revealed no significat difference among groups in terms of LA diameters, IVS diameter, LVPW diameter, LVEDD, LVESD, left ventricle mass index, Mitral E and A flow rate, left ventricle IVRT and isovolumetric contraction time (IVCT), ejection time S', E', A', EF measurements (p>0.05). However, SPAP, right ventricle IVRT, IVCT and RA diameter measurements were significantly different among groups (p<0.05) (Table 3).

**Table 3:** Comparison of inter-group transthoracic and tissue Doppler echocardiography findings

	Control Group (n:40)	Mild Stage COPD (n:35)	Severe Stage COPD (n:35)	P*
**Right atrium** (cm)	3.27±0.27^a^,^b^	3.57±0.33^a^,^c^	3.82±040^b^,^c^	**<0.001**
**Left atrium **(cm)	3.51±0.40	3.53±0.54	3.57±0.51	0.86
**IVSd** (cm)	0.99±0.14	0.99±0.16	1.01±0.13	0.96
**LVEDD** (cm)	4.52±0.40	4.60±0.37	4.60±0.52	0.66
**LVPWd** (cm)	1.03±0.15	1.02±0.12	1.02±0.12	0.88
**LVESD** (cm)	3.20±0.30	3.25±0.35	3.24±0.39	0.06
**LV EF** (%)	62.67±3.33	62.21±3.34	62.23±3.79	0.81
**SPAP** (mmHg)	18.13±5.21^d^,^e^	23.51±5.10^d^,^f^	30.80±10.20^e^,^f^	**<0.001**
**LVMI** (gr/m^2^)	98.32±20.67	108.27±30.02	105.56±24.11	0.20
**E** (m/s)	0.81±0.15	0.78±0.12	0.80±0.14	0.59
**A** (m/s)	0.85±0.15	0.83±0.15	0.84±0.12	0.87
**E′** (m/s)	0.10±0.02	0.09±0.018	0.093±0.026	0.11
**A′** (m/s)	0.104±0.021	0.105±0.018	0.106±0.030	0.93
**IVRT left** (ms)	79.60±14.24	79.05±14.43	79.32±22.77	0.99
**IVCT left** (ms)	58.01±7.08	59.26±9.47	59.37±12.91	0.80
**ET left** (ms)	357.42±47.78	274.89±38.03	278.04±54.63	0.06
**S** (m/s)	0.088±0.019	0.085±0.022	0.089±0.024	0.67
**IVRT right** (ms)	61.58±10.84a′,b′	71.54±13.9a′,c′	74.38±14.43b′,c′	**<0.001**
**IVCT right** (ms)	52.46±8.77d′,e′	59.13±12.55d′,f′	59.39±12.66e′,f′	**0.013**
**ET right** (ms)	287.54±37.44	294.57±35.93	291.82±47.14	0.74

* One-wayAnova test

Mean±SD, p<0.05, *Kruskal-Wallis Test, Median (%25–%75),p<0.05

IVSd: Diastolic thickness of interventricular septum, LVEDD: Diastolic diameter of left ventricle, LVPWd:Diastolic thickness of left ventricle posterior wall, LVESD: Systolic diameter of left ventricle, LVEF:Ejection fraction of left ventricle, SPAP:Systolic Pulmonary artery pressure, LVMI: Left ventricle mass index, MVE vel: Mitral E flow rate, MVA vel: Mitral A flow rate, IVRT: Isovolumetric relaxation time, IVCT: Isovolumetric contraction time, ET: Ejection time

a Control group-mild stage COPD (p=0.001)

b Control group–severe stage COPD (p=<0.001)

c Mild stage COPD-Severe stage COPD (p=0.008)

d Control group-mild stage COPD (p=0.005)

e Control group-severe stage COPD (p=0.001)

f Mild stage COPD-severe stage COPD (p=0.001)

a′ Control group-mild stage COPD (p=0.04)

b′ Control group-severe stage COPD (p=<0.001)

c′ mild stage COPD-severe stage COPD (p=0.664)

d′ Control group-mild stage COPD (p=0.038)

e′ Control group-severe stage COPD (p=0.029)

f′ Mild stage COPD-Severe stage COPD (p=0.998)

x: Control group-mild stage COPD (p=0.126), y: Control group-severe stage COPD (p=0.011), z: Mild stage COPD-severe stage COPD (p=0.990)

After Bonferroni adjustment, we found statistically significant differences between the RA diameter and PWD values between the study groups. For RA diameter; control – mild COPD, p=0,001; control – severe COPD, p=0,001; mild – severe COPD, p=0,008. For PWD diameter; control – mild COPD, p=0,014; control - severe COPD, p=0,026; mild – severe COPD, p=1,000.

In tissue Doppler examination, tricuspid PA was longer than the control group in both mild and severe COPD groups (p<0.001). Lateral and septal PA was similar in all three groups (p=0.85, p=0.7, respectively). The intra-right atrial EMD, atrial conduction times and electromechanical delay times are summarized in Table 4.

**Table 4:** Inter- and intra-atrial EMDs measured by tissue Doppler imaging

	Control Group (n:40)	Mild Stage COPD (n:35)	Severe Stage COPD (n:35)	p*
**Lateral PA (ms)**	54.8±8.7	55.9±12.6	56.2±14.4	0.85
**Septal PA (ms)**	41.7±8.3	42.5±9.0	43.6±11.7	0.70
**Tricuspid PA (ms)**	25.2±5.8^a^,^b^	31.8±8.1^a^,^c^	32.6±8.8^b^,^c^	<0.001
**Intra-left atrial EMD** **(ms)**	13.1±6.7	13.4±7.1	12.6±6.0	0.46
**Intra-right atrial EMD** **(ms)**	16.4±7.3^d^,^e^	10.7±5.8^d^,^f^	11.0±7.7^e^,^f^	<0.001
**Interatrial EMD (ms)**	29.5±9.1^x^,^y^	24.1±9.1^x^,^z^	23.9±11.1^y^,^z^	<0.001

* OnewayAnova

COPD: Chronic Obstructive Pulmonary Disease, PA: Delay from the onset of P-wave on ECG to the onset of A-wave on tissue Doppler recordings, EMD: Electromechanical delay

a Control group-mild stage COPD (p=0.001)

b Control group-severe stage COPD (p=<0.001)

c mild stage COPD-severe stage COPD (p=0.999)

d Control group-mild stage COPD (p=0.002)

e Control group-severe stage COPD (p=0.003)

f Mild stage COPD-severe stage COPD (p=1.000)

x Control group-mild stage COPD (p=0.057)

y Control group-severe stage COPD (p=0.042)

z Mild stage COPD -severe stage COPD (p=0.906)

### Correlation analysis

In mild stage COPD regardless of age, gender, height and weight; a moderately positive relationship was found between Pmax (r = 0.529, p = 0.002), PWD (r = 0.419, p = 0.002) and RA diameter. Pmax (r = 0.359, p = 0.047) and PWD (r = 0.556, p = 0.001) were also moderately correlated with SPAP. There was a weak correlation between PWD and tricuspid PA (r = 0.397, p = 0.027).

In severe stage COPD, Pmax and PWD had a strong positive correlation (r=0.756, p=<0.001). Pmax and LVEF exhibited a significant negative correlation (r=-0.411, p=0.022). We also used partial correlation analysis to control the effects of age and body mass index and detected that PWD and RA values had a statistically significant positive correlation (r=0.871, p=0.020) in the patients with COPD.

In mild stage COPD, PA had a negative correlation with LVEF (r=-0.498, p=0.008). Similarly, Septal PA values also had a significant negative correlation with LVEF (r=-0.487, p=0.005. Also; intra-left atrial EMD values correlated positively with LA (r = 0.418, p = 0.019). There was a positive correlation between tricuspid PA and RA too (r=0.408, p=0.028).

The analysis of severe stage COPD showed a positive correlation between atrial delay times. The intra-left atrial EMD values of severe stage COPD had a positive correlation with LA (r=0.342, p=0.022). There was a positive correlation between tricuspid PA and RA too (r=0.408, p=0.023). There was a negative correlation between tricuspid PA and spirometric test of FEV1/FVC (r=-0.322, p=0.003). Again in severe stage COPD, SPAP and FEV1 (r=-0.522, p=<0.001), and SPAP and FEV1/FVC (r=-0.344, p=0.002) had negative correlations.

## Discussion

Supraventricular arrhythmias are common in COPD compared to the normal population. Studies have shown that PWD and Pmax can be prolonged due to many different clinical conditions, and these are predictors of AF,^18^–^21^. In a prospective study evaluating signal average ECG and P wave times, Aytemir et al. followed paroxysmal AF patients for 6 months after pharmacological or electrical cardoversion. At the end of this period, P wave duration of patients with AF recurrence was longer than patients with normal sinus rhythm^22^. In a study conducted by Yildirim et. al. in 2012, patients with white coat hypertension were compared with normotensive and essential hypertensive patients; Pmax and PWD values of white coat hypertension and essential hypertensive patients were significantly higher than normotensive patients^23^. Yıldırım et al. also reported that the duration of PWD and Pmax increases as the age increases and this may be related to changes in the left atrium associated with aging^24^.

In our study, the diameter of RA was increased significantly in both COPD groups compared to the control group. Due to the increased RA pressure and diameter in COPD patients, there was an increased atrial depolarization time and increased changes between Pmax and Pmin values, and therefore increased PWD in COPD patients. In addition, we found a statistically significant relationship between PWD and RA in patients with COPD, regardless of age and body mass index. In this context, RA dilation in COPD is thought to cause increased PWD. In a study comparing P wave duration of COPD and healthy individuals; significantly higher Pmax, Pmin and PWD values were found in COPD than healthy individuals^25^. In our study, unlike the previous one, Pmax and Pmin values were statistically similar in COPD and control groups; however, PWD was prolonged compared to the healthy control group, although it was similar in both COPD groups. Although RA diameters were significantly different between mild and severe stage COPD, there was no difference between PWD values. P wave duration and PWD values showed strong positive correlation in COPD groups. However, we believe that Pmin values cannot be used as an AF risk predictor in COPD because Pmin values have shown similar results in both COPD groups and the control group.

Baseline AEMD values of 249 people with sinus rhythm were recorded in the study of De Vos et al. during the first checkup at the end of the second year, AF developed in 15 patients (6%) with long AEMD. In addition, the subgroup analysis revealed a higher prevalence of COPD in patients who developed AF. Researchers concluded that AEMD can be used as a marker for AF development^23^.

In our study, although similar lateral PA and septal PA values were found in mild and severe stage COPD patients, there was a significant increase in tricuspid PA measurements in both patient groups compared to the healthy control group. In another study conducted in 2012, supporting our study, patients had prolonged tricuspid PA measurements compared to healthy individuals, despite similar lateral PA and Septal PA values among COPD patients and healthy individuals^25^. However, unlike ours, COPD was not staged in this study. In our study, tricuspid PA values were statistically different between mild and severe stage COPD, and tricuspid PA times increased depending on the severity of COPD. Another result of our study is the strong positive correlation between COPD severity and RA diameter and tricuspid PA. Considering previous studies, increased RA diameters and tricuspid PA values, which show a progressive upward trend depending on the COPD stage, can be considered as a risk indicator for AF. In parallel with previous studies, we found a significant positive correlation between intra-atrial atrial EMD and LA diameters, although our LA diameter values were within normal limits.

In recent study conducted by Caglar et. al., despite the presence of similar Lateral PA and Septal PA values between COPD, the patients had prolonged tricuspid PA measurements than the healthy individuals. It was reported that prolonged tricuspid PA was positively correlated with RA area and SPAP, and had a negative correlation with FEV1 measurements^25^. In our study, the high SPAP values in severe stage COPD group showed a significant negative correlation with COPD stage determinant FEV1 values and FEV1/FVC values. In severe COPD, negative FEV1/FVC ratio had prolonged the tricuspid PA. Together with right ventricle enlargement, right ventricle hypertrophy leads to tricuspid annulus enlargement, functional tricuspid insufficiency and RA dilatation. Re-modelling of RA disrupts and slows down the electrical conduction paths as a result of all these factors. This situation can explain the prolongation reason of tricuspid PA these circumstances. High PAB levels can increase secondary atrial tension; cause delays in atrial depolarization and therefore extend the AEMD^26^–^29^.

In case of a clinical condition that is related to right cardiac cavities and cause prolonged tricuspid PA, such as COPD, EMD between left and right conduction times would decrease due to the right conduction delay^30^. And this will lead to prolonged tricuspid PA values and shortened Intra-right atrial EMD and Inter-atrial EMD values in COPD. These findings may play an important role in determining the AF development risk. The early prediction of AF in COPD and elimination of triggering factors of AF are very important for the prognosis of these patients.

Small study population, cross-sectional study design and no follow-up of the cases are the major limitations of our study. In addition, another limitation is the absence of computer-assisted calculation system for the measurement of Pmax and Pmin times in echocardiographic assessment. Not using 48-hour rhythm holter analysis for the exclusion of AF and arrhythmia patients is another limitation.

## Conclusion

We found increased PWD and tricuspid PA values in patients with COPD. In addition, inter-atrial and intra-atrial EMD decreased in the same patient group. All these measurements were correlated with COPD severity. We concluded that the combination of PWD and tricuspid PA may be helpful to predict the AF in patients with COPD.

We guarantee that; All authors who have participated in the work take responsibility for the manuscript which they read and approved, and which has never been published or submitted for publication elsewhere.
